# **Modified kraft lignin as sustainable consolidants for waterlogged archaeological wood**

**DOI:** 10.1038/s41598-025-21767-z

**Published:** 2025-10-29

**Authors:** Ahmet Erdem Yazici, Jakub Brózdowski

**Affiliations:** Department of Chemical Wood Technology, Poznań University of Life Sciences, Poznań, Poland

**Keywords:** Lignin modification, Archaeological wood, Sustainable consolidation, Waterlogged wood conservation, Bio-based materials, Chemistry, Engineering, Environmental sciences, Materials science

## Abstract

This study evaluates lightened kraft lignin: modified via acetylation and encapsulation, as a sustainable consolidant for waterlogged archaeological pine (*Pinus* sp.) from the Biskupin site (Poland). Lignin was acetylated with acetic anhydride/pyridine and, in a separate route, converted into colloidal capsules by tetrahydrofuran/water self‑assembly; both modifications were characterized and then applied to waterlogged archaeological wood by 2‑week immersion. Effectiveness was assessed by Fourier‑transform infrared spectroscopy (FTIR), scanning electron microscopy (SEM), colourimetry, linear dimensional change (LDC), anti‑shrink efficiency (ASE), and water‑uptake tests. Acetylated lignin penetrated more deeply and reduced water uptake (from > 240% to ~ 96%) with moderate improvements in dimensional stability; encapsulated lignin showed limited penetration but lowered shrinkage (tangential: from 6.78 to 4.21%; radial: from 3.36% to 2.37%) through a surface‑barrier effect and was better in preserving the natural colour. These results indicate that modified kraft lignin, particularly the encapsulated formulation, can complement or partially replace petroleum‑based consolidants and motivate further optimization of capsule size/distribution to enhance mechanical performance and treatment uniformity.

## Introduction

Conservation of waterlogged archaeological wood presents significant challenges. This wood is typically preserved in anaerobic environments where microbial degradation is slowed, allowing lignin, more resistant compared to cellulose, to remain dominant polymer. Excavation and exposure to air, often results in rapid moisture loss which can trigger irreversible collapse and shrinkage. The immediate aim of treatment is to stabilize the structure and limit deformation caused by drying while maintaining natural appearance and allowing retreatability^1,2^. Historically and currently, consolidants include polyethylene glycol (PEG), melamine‑formaldehyde (MF), sugars/sugar alcohols, and organosilicons. PEG is a long‑standing benchmark but may increase hygroscopicity and alter appearance depending on molecular weight and drying schedule, reported anti‑shrink efficiency (ASE) values vary with degradation state and processing (e.g., Scots pine with PEG 400/4000 can achieve high ASE in radial direction for highly degraded sapwood, but performance depends strongly on drying strategy)^3,4^. Organosilicon routes have been proposed as reversible/retreatable options and shown to stabilize dimensions effectively^5,6^. Recent work on reversibly cross‑linked polymers reports high dimensional stability (ASE up to ~ 96%), highlighting a push toward reversible chemistries, while sustainability concerns further motivate transition away from petroleum‑derived systems^7–10^.

Within this evolving landscape, bio‑based alternatives have gained attention. Biological or bio‑derived materials explored for conservation include nanocellulose and chitosan protective coatings, lignin‑based systems such as lignin nanoparticles, and other bio‑derived polymers. Protective cellulose nano-fibres (CNF)/cellulose nanocrystals (CNC)/chitosan coatings have shown barrier and optical benefits, while lignin nanoparticles have recently been proposed and tested as consolidants for waterlogged wood^11–13^. Lignin is particularly attractive because it is abundant, compatible with the wood matrix, and exhibits antimicrobial, antioxidant, and UV‑screening properties, yet it remains relatively underutilised to its ~ 50 Mt/year production^14–16^.

A practical limitation of kraft lignin in conservation is colour^17^: conjugated chromophores darken treated surfaces, which can be unacceptable aesthetically. Chemical/light‑management strategies therefore target both performance and optical properties. Acetylation reduces hydrogen bonding and improves solubility/dispersion; when followed by solvent‑exchange self‑assembly, it yields light‑scattering colloids that visually lighten lignin while enabling controlled deposition within wood porosity^18^. Parallel bio‑inspired routes of consolidation of waterlogged archaeological wood use in situ polymerization of phenolics (e.g., isoeugenol), offering greener chemistries for consolidation^19^.

The present work investigates two lightened kraft lignin formulations: acetylated lignin and encapsulated (self‑assembled) colloids as consolidants for waterlogged archaeological pine (*Pinus* sp.) from Biskupin.

## Experimental

### Materials

#### Materials

Archaeological pine wood (*Pinus* sp.) was sourced from Biskupin, a major European site in northern central Poland dating from the late Bronze and early Iron Age. The wood, submerged for ~ 2700 years, contained 37.8% holocellulose, 32.0% cellulose, 40.6% lignin, and 6.0% mineral substances. Samples were cut to 2.5 × 2.5 × 1 cm and stored in distilled water to prevent drying damage before treatment.

#### Chemicals

Kraft lignin (Merck) was used due to its commercial availability. Lignin specification given by Merck: molecular weight of ~ 10,000, sulphur content up to 4%, pH 10.5 (3 wt%). Tetrahydrofuran, pyridine, and ethyl acetate (Alfachem) were used for the modification of lignin; distilled water was also used throughout the process.

### Lignin modification

#### Acetylation

10 g of kraft lignin were mixed with acetic anhydride/pyridine (1:1) in a 1:5 ratio, stirred until dissolved and kept in the dark at 50 °C for 48 h. The product was filtered (6 μm), washed with distilled water until the acetic anhydride odour disappeared and then dried under vacuum.

#### Encapsulation

Following Qian et al.^18^, acetylated lignin was dissolved in THF and slowly added to distilled water by sonication and stirring. The resulting suspension of the lignin sphere was evaporated using a rotary evaporator and stored in sealed containers at room temperature.

### FTIR analysis

To evaluate chemical changes associated with acetylation, encapsulation, and wood impregnation, Attenuated Total Reflectance Fourier Transform Infrared (ATR-FTIR) spectroscopy was used. Spectra were collected for both modified lignin and archaeological wood. Samples were placed directly on the diamond ATR crystal and pressed with the built-in clamp to ensure close contact. Spectra were acquired on a Bruker spectrometer and processed in OPUS software. After baseline correction and denoising, spectra were normalized by max-peak scaling (wood: 1800–600 cm⁻¹; lignin: 4000–600 cm⁻¹); 16 scans were co-added per specimen to obtain the final spectrum.

### SEM analysis

Scanning Electron Microscopy (SEM) was used to detect morphological changes in raw, lightened, and encapsulated lignin, as well as for treated wood. During the imaging process, the Zeiss EVO 40 electron microscope was used.

### Wood impregnation

The samples were impregnated by immersion in a modified lignin solution. Samples immersed in a pure solvent were used as the controls. Acetylated lignin was dissolved in ethyl acetate at 10%, 20%, and 30%, although complete dissolution was not achieved at 30%. Encapsulated lignin was suspended in water, but particle sedimentation occurred already at 20%, so only 10% and 20% dispersions were used. It should be noted that the 30% acetylated and the 20% encapsulated formulations did not represent fully homogeneous systems due to partial sedimentation; nevertheless, they were included in the study as the maximum workable concentrations. All samples were immersed at room temperature for two weeks to ensure effective impregnation; two-week impregnation was suggested by Łucejko et al.^17^ as reference time of impregnation. Impregnation and properties were tested in three repetitions *n* = 3.

Sample labels are shown in the Table 1.

**Table 1:** Description of the samples.

Sample	Solvent	Lignin treatment	Concentration
ET0	Ethyl acetate	–	–
ET10	Ethyl acetate	Acetylated	10%
ET20	Ethyl acetate	Acetylated	20%
ET30	Ethyl acetate	Acetylated	30%
W0	Water	–	–
W10	Water	Self-Assembly	10%
W20	Water	Self-Assembly	20%

To detect dimensional changes in archaeological wood after impregnation, pins were attached to the samples. The samples were then air dried for 4 days until they reached a constant weight under controlled conditions (50% RH and 20 °C).

### Dimensional stability

The dimensional changes of archaeological wood samples were measured by reference pins placed according to Łucejko et al. ^17^. Dimensional stability in wood is evaluated by using both linear dimensional change (LDC) and anti-shrink efficiency (ASE). LDC measures the percentage change in the sample’s size followed by the treatment.

Linear dimensional change Eq. (1) and anti-shrinkage efficiency Eq. (2) were calculated as follows:

1 $$\:Linear\:Dimensional\:Change\:\left(\%\right)=\:\frac{(Dimension\:After\:\left(Rad/Tan\right)-Dimension\:Before\:(Rad/Tan\left)\right)}{Dimension\:Before\:(Rad/Tan)}\times\:\:100$$

2 $$\:Anti-Shrink\:Efficiency\:\left(\%\right)=\:\frac{Su-\:St}{St}\times\:\:100$$

where Su: Shrinkage percentage of the untreated sample, St: Shrinkage percentage of the treated sample.

A higher ASE value indicates more effective treatment, as it shows a greater reduction in shrinkage. Values close to 100% indicates higher dimensional stabilization, while negative values suggest increased shrinkage compared to the control samples.

### Water uptake

Water uptake was measured to assess the hydrophobic effectiveness of acetylated and encapsulated lignin treatments by comparing treated and control archaeological wood samples. Dried samples (W₀) were weighed, immersed in distilled water for 24 h at room temperature, then reweighed (W₁) after gently wiping off surface water. Water uptake was calculated using the Eq. (3)

3 $$\:Water\:Uptake\:\left(\%\right)=\:\frac{\text{W}_{1}-\text{W}_{0}}{\text{W}_{0}}\times\:\:100$$

Lower water uptake indicates increased hydrophobicity and effective moisture resistance, while higher values suggest the material remains hygroscopic.

### Colorimetric analysis

To detect the colour change in the archaeological wood before and after the treatment, colorimetric analysis was conducted. The samples were tested for colour change with spectrophotometer before and after the treatment. The colour measurements were recorded using a Datacolor 600 spectrophotometer. The colour change in the CIE Lab system was calculated according to the Eq. (4)

4 $$\:\varDelta\:E=\sqrt{(\varDelta\:{L)}^{2}+(\varDelta\:{a)}^{2}+(\varDelta\:{b)}^{2}}\:$$

Obtained results were used to calculate ∆E. The colour difference is represented by the variable ∆E, which is calculated based on the achromatic coordinate L that represents the brightness of the colour (where L = 100 is white and L = 0 is black), and the chromatic coordinates a and b that describes the colour.

∆E is a colorimetric parameter that quantifies the perceptible difference between two colours in a three-dimensional colour space. A higher ∆E value indicates a larger visual deviation, while values below 1 are generally considered undetectable by the human eye.

## Results and discussion

### Lignin characterisation

**Fig. 1:**
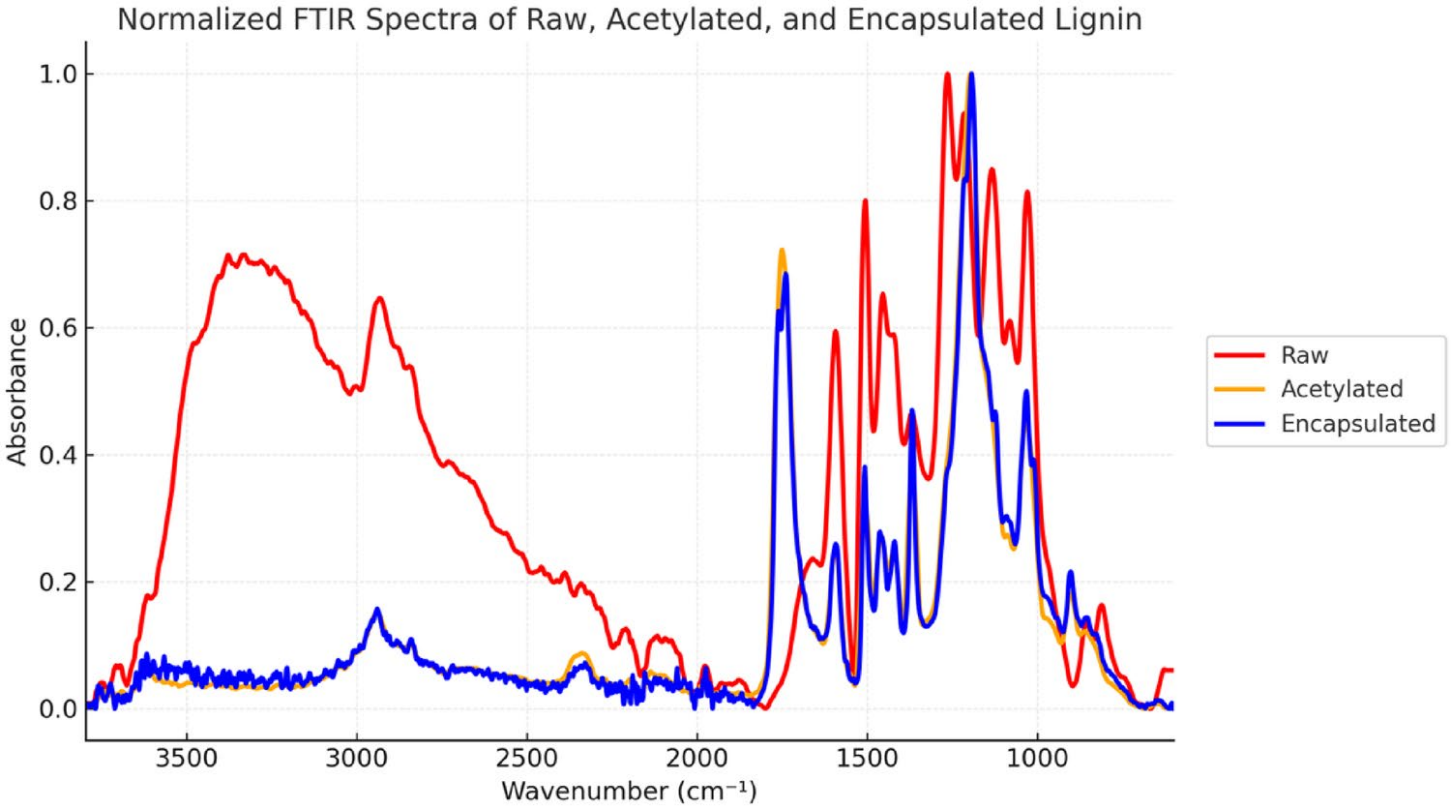
FTIR spectrum of raw lignin, acetylated lignin and encapsulated lignin.

Normalized FTIR spectra (Fig. 1) were used to compare absorbance intensities across lignin samples. The peak at ~ 1745 cm⁻¹, corresponding to C = O stretching of ester bonds, indicates successful acetylation of aliphatic and phenolic –OH groups^20^. No new bands or significant shifts were observed between acetylated and encapsulated lignin, suggesting that encapsulation involves physical, not chemical, interactions. A reduction in the broad –OH stretching band (3300–3400 cm⁻¹) further confirms acetylation and may also reflect lignin purification via removal of carbohydrate residues from kraft processing.

**Fig. 2:**
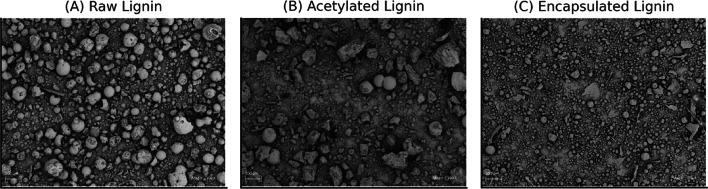
SEM pictures of raw (**A**), acetylated (**B**) and encapsulated (**C**) lignin (100x Magnification).

The morphology of raw, acetylated, and encapsulated lignin was examined via SEM (Fig. 2A–C), revealing clear structural changes after modification. Figure 2A shows that raw lignin has an irregular, particulate surface with visible pores, micro-voids, and fragmented areas, reflecting its naturally heterogeneous microstructure.

Figure 2B shows a smoother, more compact surface after acetylation, compared to the raw lignin Fig. 2A. Particles appear denser, with sharper edges and flattened areas, suggesting bonding between acetyl and –OH groups. This modification hydrophilizes the lignin and improves structural uniformity compared to raw lignin. Figure 2C shows that encapsulated lignin forms a more compact and uniform structure, smaller than acetylated lignin. Pores are significantly reduced, and surface roughness is lower, likely due to the enclosure of lignin within a matrix. Encapsulation appears to enhance particle binding and improve physical stability.

The colour difference values of acetylated and encapsulated lignin samples were calculated based on raw lignin, which has the following basic values: L = 32.79, a = 9.63, b = 15.97, C = 18.64, h = 58.91. The results of colour analysis are presented in Table 2.

**Table 2:** Average colour changes of acetylated (ET) and encapsulated (W) lignin in comparison with Raw kraft lignin.

Parameter	Raw kraft lignin	ET	W
L*	32.79	17.15	21.63
a*	9.63	− 2.71	− 0.22
b*	15.97	9.97	16.28
C*	18.64	8.22	14.90
H*	58.91	6.29	6.45
ΔL*	–	− 15.64	− 11.16
Δa*	–	− 12.34	− 9.85
Δb*	–	− 6.00	0.31
ΔE*	–	20.81	14.89
Picture			

Colour data given in the Table 2 refers to the pre-impregnated state of acetylated (ET) and encapsulated (W) lignin and are important for comparing the optical properties of both groups. The values show that acetylated lignin is slightly darker (L = 17.15) and duller (C = 8.22), while encapsulated lignin is lighter (L = 21.63) and more saturated (C = 14.90).

It was observed that the encapsulation process preserved the original colour properties of the lignin better. Moreover, the total colour change value (ΔE) was higher with acetylated lignin (20.81). This indicates a stronger colour change during the acetylation process, while the change was more moderate in encapsulated lignin (14.89). This difference is possibly due to the fact that acetylation process provides direct molecular interactions while encapsulation process provides more surface physical structuring. For conservation practice, the brightening effect of encapsulated lignin may be particularly advantageous, as darkening of surface is often seen as a throwback by curators.

### Evaluation of wood impregnation with modified lignin

To assess lignin efficiency and penetration, samples were divided into outer, inner, and core regions. Scans of the outer region were taken at the surface of the sample, scans of inner region were taken from the middle of the sample, in-between, surface and the core of the sample cross-section, scans from the core were taken and the centre of the sample. Ethyl acetate- and water-immersed controls were used for the ET and W groups, respectively. The 1745 cm⁻¹ peak (C = O stretching of ester groups) served as a marker of acetylated lignin penetration, absent in controls and thus indicating successful impregnation.

The outer layer, directly exposed to treatment, is expected to show the most pronounced spectral changes. The inner layer reflects intermediate diffusion, while the core represents the most difficult region to reach due to structural resistance.

**Fig. 3:**
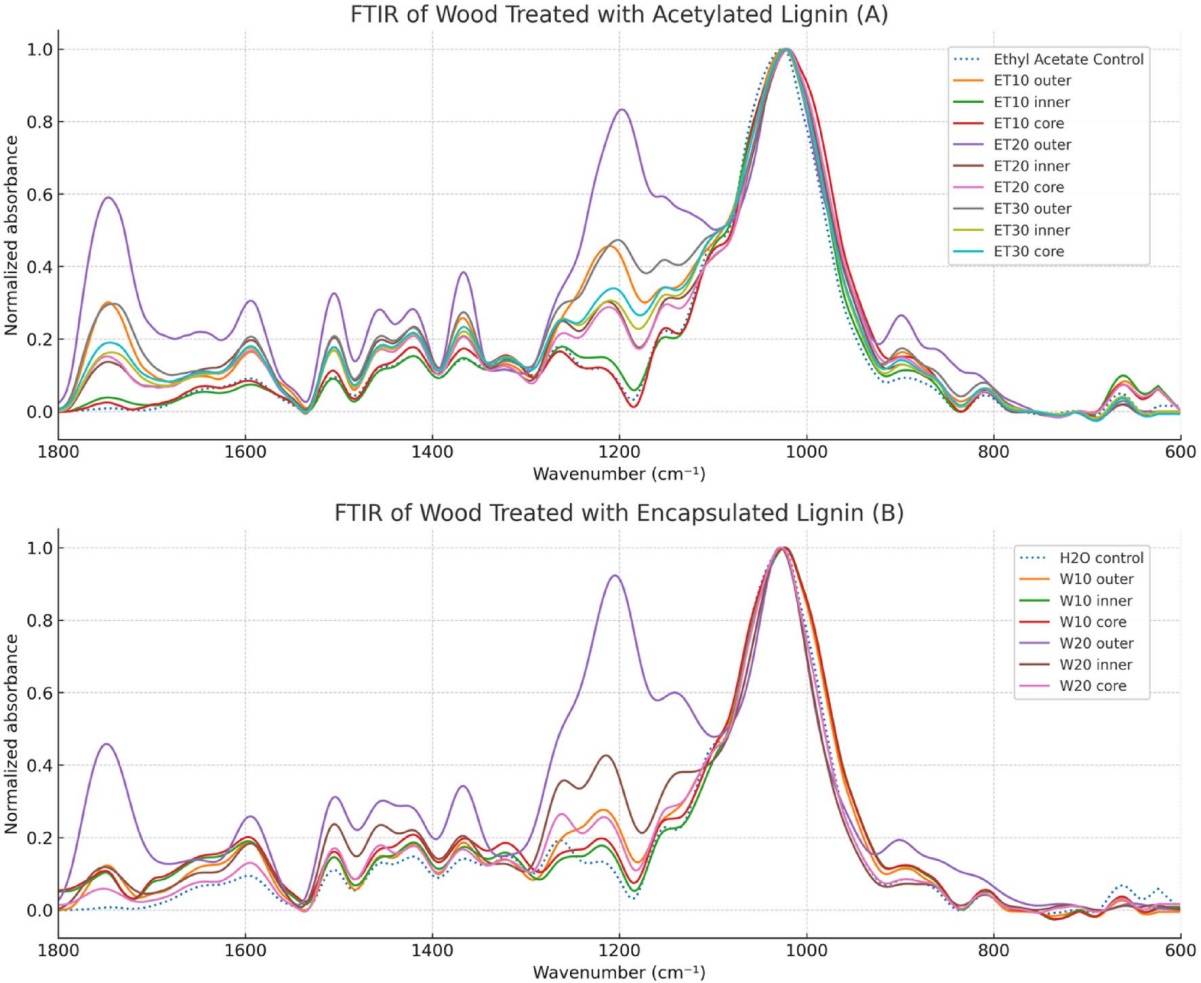
Normalized FTIR spectra of lignin impregnated archaeological wood.

The findings are presented in the Fig. 3.

Analysis of the FTIR spectrum for samples ET10, ET20 and ET30 (Fig. 3A) resulted in the following observations. The ET10 sample exhibits a strong acetylation peak at 1745 cm⁻¹ in the outer layer, whereas the inner and core layers display peak intensities similar to the control, indicating limited penetration of 10% acetylated lignin. In contrast, the ET20 sample shows more intense 1745 cm⁻¹ signals across all regions, including the inner and core layers, suggesting enhanced diffusion. The ET30 sample presents the most prominent and consistent acetylation peak throughout the entire cross-section, with negligible differences in intensity between the layers. This implies a more uniform and deeper penetration of the impregnation solution.

In general, as the concentration increases, the penetration from the outer layer to the core also increased. An indicator of successful lignin penetration toward the core of the sample is the appearance of a peak at approximately 1745 cm⁻¹, corresponding to the acetylation of phenolic hydroxyl groups in the modified lignin^20^.

The W10 (Fig. 3B) samples exhibit relatively uniform 1745 cm⁻¹ peaks across the cross-section; however, the overall intensity of this peak is low. This suggests that although penetration from the outer layer toward the core occurred, the amount of acetylated lignin reaching the core was limited.

In W20 samples, a strong 1745 cm⁻¹ peak is observed in the outer layer, with a gradual decrease in intensity toward the core. Nevertheless, the acetylation peak remains detectable even in the core region, in contrast to the control sample, indicating deeper diffusion.

In general, compared to the ET group, which showed a consistent improvement in lignin penetration from the surface to the core regions with increasing concentration, the W group exhibited a similar trend, however to a lesser extent. Depth of penetration was markedly lower in the W group compared to the ET group.

Due to the better results achieved during the modification process, ET30 and W20 treatments were chosen for SEM analysis. A significant difference was observed between the treatments.

**Fig. 4:**
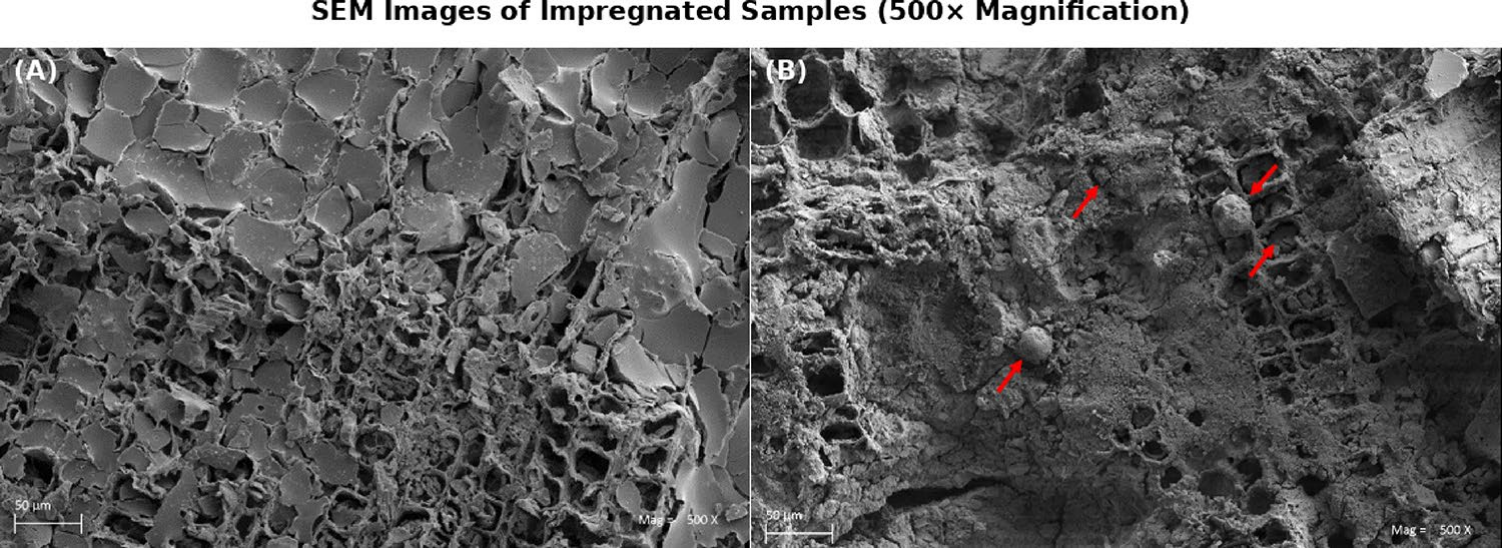
SEM pictures of wood impregnated with acetylated (**A**) and encapsulated (**B**) lignin (500x magnification). Arrows in picture (**B**) Show lignin capsules.

For the sample treated with ET30 (Fig. 4A), film-like layer of lignin was observed uniformly covering the sample surface. This outcome supports that acetylated lignin interacts effectively and homogeneously with the wood surface. The formed film layer is thought to potentially facilitate a deeper penetration and overall impregnation efficiency.

The sample treated with encapsulated lignin (Fig. 4B), compared to the one treated with acetylated lignin (Fig. 4A), reveals a distinct morphology characterized by spherical particles distributed on the surface unevenly—single or clustered capsules on the surface and some capsules partially buried in the vessels can be seen on the image, pointed by the arrows (Fig. 4B).

The depth of penetration depended strongly on the impregnation medium. In the case of acetylated lignin, ethyl acetate acted as a good solvent, producing a homogeneous solution that facilitated diffusion of lignin molecules into the wood structure. In contrast, encapsulated lignin was dispersed in water as colloidal particles of larger size, which reduced its mobility and thus limited its penetration into the cell walls. Consequently, acetylated lignin (ET) penetrated deeper and established stronger interactions within the wood matrix, whereas encapsulated lignin (W) mainly formed a surface-level barrier.

Archaeological objects often need a re-treatment, and one the role of consolidant is to give that possibility^2,19^. Wood cells after conservation needs to be able to absorb new portion of consolidant. Open structure of lumens filled with encapsulated lignin gives chance for re-treatment, in contrast, film layer formed by acetylated lignin may prevent re-treatment of the object.

### Colour analysis

The results of colour change analysis are as follows: with increasing lignin concentration in the ET group (Table 3), surface darkening intensified, especially in ET20 and ET30, which showed a nearly uniform black tone. Cross-sections of ET30 confirmed deep penetration, with colour reaching the core, indicating effective diffusion of acetylated lignin at higher concentrations.

In contrast, W group samples (Table 3) remained lighter despite increased concentration. Internal areas resembled the control (W0), suggesting encapsulated lignin mainly adhered to the surface, with limited penetration—consistent with SEM observations.

**Table 3:** The colorimetric analysis of the samples.

Parameter	ET0	W0	ET10	ET20	ET30	W10	W20
L	39.11	35.84	35.78	26.98	19.65	43.42	57.23
a	4.08	3.22	8.39	5.08	3.88	4.71	5.91
b	14.21	11.82	17.56	11.52	5.55	19.13	23.62
C	14.79	12.26	19.48	12.59	6.78	19.70	24.35
H	73.96	75.00	64.31	66.15	54.81	76.18	75.94
ΔL	–	–	− 3.33	− 12.13	− 19.46	7.58	21.39
Δa	–	–	4.31	1.00	− 0.21	1.49	2.69
Δb	–	–	3.35	− 2.70	− 8.66	7.31	11.79
ΔE	–	–	6.39	12.46	21.30	10.63	24.57
Picture							

Number after group symbol (ET—acetylated lignin, W—encapsulated lignin) indicates the concentration of modified lignin used in the treatment.

Colorimetric analysis) showed a marked decrease in L* values from ET0 (39.11) to ET30 (19.65), indicating progressive surface darkening, consistent with macroscopic observations. Chroma (C*) also declined with increasing acetylated lignin concentration, reflecting reduced colour saturation. All ET samples exhibited ΔE values above 5, exceeding the commonly accepted visibility threshold (ΔE > 3–5), confirming that the colour changes are perceptible to the naked eye. In contrast, the encapsulated lignin group showed increased L* and C* values, suggesting lighter and more saturated surfaces. High ΔE values in W10 (10.63) and particularly W20 (24.57) confirm pronounced and visually favourable brightening, giving the treated wood a lighter, slightly yellowish appearance. Łucejko et al.^17^ similarly reported darkening after ethyl acetate lignin impregnation due to the natural colour of lignin. In this study, encapsulation produced lighter tones compared to acetylation. The preservation of wood’s natural appearance is an important factor when selecting a consolidant, and Broda and Hill^10^ highlighted colour retention as a clear advantage of certain consolidants.

### Dimensional stability

Dimensional stability was assessed using linear dimensional change (LDC) and anti-shrink efficiency (ASE), calculated for both tangential (T) and radial (R) directions. The results are presented in the Table 4.

**Table 4:** The dimensional stability evaluation of the samples (mean ± SD).

Group	Direction	Linear dimensional change [%]	Shrinkage [%]	Anti-shrink efficiency [%]	Water uptake [%]
ET0	T	− 5.05 ± 3.76	5.05 ± 3.76	–	241.68 ± 113.02
	R	− 4.83 ± 3.39	4.83 ± 3.39	–	
ET10	T	− 6.82 ± 3.30	6.82 ± 3.30	− 35.00 ± 65.42	102.10 ± 36.95
	R	− 2.65 ± 1.26	2.65 ± 1.26	45.08 ± 26.05	
ET20	T	− 5.83 ± 2.20	5.83 ± 2.20	− 15.56 ± 43.59	95.89 ± 4.64
	R	− 3.44 ± 1.32	3.44 ± 1.32	28.79 ± 27.25	
ET30	T	− 4.01 ± 7.18	4.01 ± 7.18	20.58 ± 14.22	128.25 ± 31.98
	R	− 3.47 ± 2.20	3.47 ± 2.20	28.25 ± 45.59	
W0	T	− 6.78 ± 0.60	6.78 ± 0.60	–	103.43 ± 14.26
	R	− 3.36 ± 0.99	3.36 ± 0.99	–	
W10	T	− 7.43 ± 1.42	7.43 ± 1.42	− 9.49 ± 20.92	215.31 ± 150.70
	R	− 2.48 ± 1.52	2.48 ± 1.52	26.28 ± 45.12	
W20	T	− 4.21 ± 1.48	4.21 ± 1.48	38.00 ± 21.82	271.85 ± 12.68
	R	− 2.37 ± 1.77	2.37 ± 1.77	29.45 ± 52.63	

Number after group symbol (ET—acetylated lignin, W—encapsulated lignin) indicates the concentration of modified lignin used in the treatment.

Values represent the mean of three samples. Tukey HSD test comparing treated groups to their respective controls (ET vs. Ethyl Control; W vs. H₂O Control) did not reveal statistically significant differences (*p* > 0.05). Although some treatments (e.g., ET30 for water uptake, W20 for shrinkage) showed trends towards improved dimensional stability, these effects were not significant given the variability among replicates. Increasing the number of replicates would likely improve statistical power; however, archaeological material is scarce and sample numbers are inherently limited. This study should therefore be regarded as a preliminary investigation, intended to highlight the potential of lignin as a sustainable and biocompatible consolidant.

Dimensional stability is a critical parameter in evaluating consolidants for waterlogged archaeological wood, as excessive shrinkage during drying leads to irreversible deformation. In this study, acetylated lignin treatments (ET group) showed moderate reductions in water uptake and shrinkage compared with untreated controls, particularly in the tangential direction. Encapsulated lignin (W group), although less penetrating, provided an effective surface barrier that decreased tangential shrinkage from 6.78% to 4.21% and radial shrinkage from 3.36% to 2.37%. This result shows that at surface deposition of capsules can counteract capillary forces during drying, even without deep penetration.

Our results are comparable to those reported for bio-based polysaccharides. Villani et al.^21^ found that sodium alginate provided limited stabilization, with maximum ASE values of ~ 40%, which fell below the 75% threshold generally considered effective for conservation. Nonetheless, alginate’s performance was improved when slow drying allowed better polymer retention. Similarly, in our study, encapsulated lignin benefited from its colloidal nature, forming surface barrier that reduced collapse without altering colour significantly.

When compared to traditional consolidants such as polyethylene glycol (PEG), the differences become clearer. Majka et al.^3^ reported ASE values often exceeding 70–90% for PEG 2000 treatments on Scots pine, confirming PEG’s strong bulking efficiency. However, PEG also introduces significant drawbacks, including increased hygroscopicity at RH > 80% and susceptibility to oxidative degradation. Lignin-based systems, while less efficient in shrinkage prevention, offer advantages in sustainability and potential compatibility with the wood’s natural chemistry.

Water uptake is crucial in assessing the hydrophobicity of the material. Within the control samples it is clearly visible that ethyl acetate control samples were exhibiting higher water uptake when comparing to the water controls. Higher hydrophilicity is most certainly cause by removal of hydrophobic extractives by organic solvent, namely ethyl acetate. Among ET samples, ET20 showed the lowest water uptake (96%), indicating effective hydrophobicity at this lignin concentration. In ET30, higher lignin content likely hindered penetration, resulting in increased water uptake. In contrast, W20 had the highest uptake (272%), exceedingly even the control. Despite high water absorption in both W samples, ASE remained high, possibly because the capsules, while absorbing water, acted as physical fillers, buffering against shrinking and swelling.

Taken together, these findings suggest that modified lignin, particularly in encapsulated form, may not yet reach the dimensional stabilization levels achieved with PEG, but present a promising direction for eco-friendly alternatives. Improvements in penetration depth and distribution, as well as further tailoring of colloid size and stability, could significantly enhance their anti-shrink efficiency while avoiding the long-term risks associated with PEG.

## Conclusion

Modified kraft lignin was successfully acetylated and encapsulated, producing materials suitable for the conservation of waterlogged archaeological wood. Acetylated lignin demonstrated deeper penetration and stronger wood integration, while encapsulated lignin enhanced dimensional stability through a barrier effect near the sample surface. Despite higher water uptake, encapsulated treatments maintained structural integrity and preserved a lighter appearance. These results highlight the potential of kraft lignin, particularly in encapsulated form, as a sustainable alternative to petroleum-based consolidants. Future work should focus on optimizing capsule size and dispersion to improve treatment uniformity and mechanical performance.

## Data Availability

The datasets generated during the current study are available from the corresponding author upon reasonable request.
